# Somatosensory Testing in Pediatric Patients with Chronic Pain: An Exploration of Clinical Utility

**DOI:** 10.3390/children7120275

**Published:** 2020-12-05

**Authors:** Anna Kersch, Panchalee Perera, Melanie Mercado, Andrew Gorrie, David Sainsbury, Tara McGrath, Phillip Aouad, Sara Sarraf, Tiina Jaaniste, David Champion

**Affiliations:** 1 Department of Pain, Sydney Children’s Hospital, Randwick, NSW 2031, Australia; anna.kersch7@gmail.com (A.K.); panchaleee@gmail.com (P.P.); melanie.anne.mercado@gmail.com (M.M.); Andrew.Gorrie@health.nsw.gov.au (A.G.); David.Sainsbury@health.nsw.gov.au (D.S.); Phillip.Aouad@health.nsw.gov.au (P.A.); ssarraf@deloitte.com.au (S.S.); Tiina.Jaaniste@health.nsw.gov.au (T.J.); 2 School of Medicine, University of New South Wales, Sydney, NSW 2052, Australia; 3 Stollery Children’s Hospital, University of Alberta, Edmonton, AB T6G 2R3, Canada; trm@ualberta.ca

**Keywords:** chronic pain, somatosensory testing, central sensitization, child, sensory, algometry, cutaneous stimuli, deep pressure

## Abstract

We aimed to evaluate the utility of clinical somatosensory testing (SST), an office adaptation of laboratory quantitative sensory testing, in a biopsychosocial assessment of a pediatric chronic somatic pain sample (N = 98, 65 females, 7–18 years). Stimulus–response tests were applied at pain regions and intra-subject control sites to cutaneous stimuli (simple and dynamic touch, punctate pressure and cool) and deep pressure stimuli (using a handheld pressure algometer, and, in a subset, manually inflated cuff). Validated psychological, pain-related and functional measures were administered. Cutaneous allodynia, usually regional, was elicited by at least one stimulus in 81% of cases, most frequently by punctate pressure. Central sensitization, using a composite measure of deep pressure pain threshold and temporal summation of pain, was implied in the majority (59.2%) and associated with worse sleep impairment and psychological functioning. In regression analyses, depressive symptoms were the only significant predictor of pain intensity. Functional interference was statistically predicted by deep pressure pain threshold and depressive symptoms. Manually inflated cuff algometry had comparable sensitivity to handheld pressure algometry for deep pressure pain threshold but not temporal summation of pain. SST complemented standard biopsychosocial assessment of pediatric chronic pain; use of SST may facilitate the understanding of disordered neurobiology.

## 1. Introduction

“Perhaps, central sensitization has been the most relevant advance of the last three decades in understanding the clinical pathophysiology of pain.”—Curatolo, 2018.

Physical examination of children and adolescents with chronic somatic (non-neuropathic) pain disorders typically includes deep pressure stimuli for focal and widespread tenderness at the primary pain site and beyond. A painful response to low intensity pressure stimulus is commonly referred to as “tenderness” and is a screening sign of low pressure pain threshold.

The most widely applied sensory measure in studies in chronic pain subjects, including in pediatric studies, is a single modality somatosensory test response: pressure pain threshold. (Supplementary Table S1). This provides limited scope for phenotypic definition and for implying regional and extended central sensitization.

It is now possible, as a result of extensive translational research, mainly in adults, to formalize office somatosensory testing (SST) also in children and adolescents. This results in more meaningful interpretations, not only in neuropathic pain disorders, but also in the much more prevalent chronic somatic pain disorders. In the latter context, inferences about peripheral neural sensitization and especially central sensitization in the individual patient are feasible.

The clinical SST process involves psychophysical stimulus–response and threshold testing of a range of cutaneous and deep pressure stimuli at pain sites and control sites. The office applications have resulted from translational research from selected applications of laboratory quantitative sensory testing (QST) [1,2]. QST has been conducted in healthy children and adolescents and in those with pain conditions (Supplementary Table S2a,b). However, laboratory QST is quite a laborious assessment process, requiring prolonged time and considerable equipment and space. It is generally not practical for day-to-day clinical use and for many clinical research projects, especially with children.

Practical multimodal bedside/office testing methods for sensory phenotyping have been developed and applied to adults [3], but not to our knowledge in children. We have developed limited clinical multimodal SST as a bedside or office-based alternative to pediatric QST [3,4]. We presented the background to and rationale for its development from laboratory-based QST of a set of somatosensory stimulus–response measures elicited from pain regions and control sites applicable to office or bedside application in pediatric patients [4]. The broad objective of this study was to improve a clinician’s ability in research and practice to characterize the sensory phenotype of chronic pain beyond “tenderness” with a view ultimately to better management. Feasibility of the test procedures have been established and extensive regional hypersensitive and hyposensitive cutaneous abnormalities and low pressure pain thresholds were observed [4,5]. Low pressure pain threshold predicted worst pain intensity [4], consistent with other pediatric pain studies (Supplementary Table S1). Furthermore, the participants and parents in this study [4] were generally interested in and appreciative of the study of the pain from a neurobiological perspective.

In the current study, we have extended the SST applications in diverse chronic pain disorders in pediatric patients to include assessments for cutaneous allodynia and facilitated temporal summation to repetitive punctate pressure and to deep pressure at the pain threshold with a view to implications of pain hypersensitivity. Pain hypersensitivity involves multiple mechanisms, including central sensitization [6], conditioned pain modulation [7], reward and motivation [8], epigenetic mechanisms [9] and neuroinflammation [10] including microglial activation [11]. Of these, a clinician has best access to signs implying central sensitization. Observations implying conditioned pain modulation are possible but are more challenging and were not included in this protocol.

There are no pediatric SST studies published so far as we can determine which have investigated by multimodal office SST for implications of central sensitization in unselected chronic pain disorders. However, Pas et al. [12] reviewed publications within their classification of hyperexcitability of the central nervous system in different chronic pain conditions in children. The authors applied the term central hyperexcitability synonymously with implied central sensitization. The 12 case-control studies included involved pressure pain threshold only or laboratory QST and are included in the Supplementary Tables S1 and S2b.

Central sensitization cannot be directly measured and there is insufficient consensus on definition from a clinical viewpoint, but there are many studies that attest to the utility of methods which imply central sensitization [2], for example in predicting outcomes of surgery [13]. After a review of the literature and being particularly influenced by the clinical studies of Arendt-Nielsen [13,14], we chose the composite of low deep pressure pain threshold and temporal summation of pain intensity from repetitive deep pressure at the pain threshold relative to control site observations to imply central sensitization.

In a subset, we evaluated cuff algometry against the more commonly used handheld pressure algometry. Despite being a validated device, even in pediatric samples [15,16], the handheld pressure algometer assesses limited tissue volume, is user-dependent and is seldom available in a clinical context [17,18]. The use of a blood pressure cuff has emerged as a valid, alternate device in adults [19,20,21,22], which is easier to apply uniformly [20] and stimulates a larger tissue volume, making it less influenced by local pain sensitivities [1,22,23,24]. Given that the cuff could be less threatening, being a familiar device to children with chronic conditions [25], it is surprising that we have found only one published cuff algometry study that included pediatric participants, and this lacked sufficient pediatric content to be meaningful [26].

### Aims and Hypotheses

The overall aim was to evaluate the clinical utility of office or bedside SST, including methods enabling implications of central sensitization, in a biopsychosocial assessment of pediatric chronic pain patients. Insight into the neurobiological dysfunction underlying chronic pain might contribute to a reduction in diagnostic uncertainty [27].

The specific primary aims and related hypotheses were firstly, to determine the frequency of hypoesthetic, hyperesthetic (including tactile allodynia) and normal responses to cutaneous and deep SST stimuli at pain sites compared to control sites, and to test the hypothesis that the SST responses would be strongly inter-correlated within the cutaneous and within the deep pressure measures and largely independent of psychological measures.

Secondly, we aimed to establish the relationship between a composite measure of implied central sensitization (determined through SST), with common clinical features of central sensitization (e.g., psychological functioning, pain-related functioning, and sleep impairment). We hypothesized that implied central sensitization would be detected in the majority of chronic pain patients and that such individuals would have more prominent clinical features commonly associated with central sensitization.

Thirdly we aimed to determine whether two SST responses believed to be associated with central sensitization, namely low deep pressure pain threshold and facilitated temporal summation of deep pressure pain, and a self-report measure of depressive symptoms are statistical predictors of pain outcomes (pain intensity and functional interference). We hypothesized that SST responses and the psychological measure would both account for a significant amount of variance.

A secondary aim was to determine, using subset analysis, whether manually inflated cuff algometry is comparable to handheld pressure algometry for the deep pressure variables.

## 2. Materials and Methods

### 2.1. Study Design

The study utilized a cross-sectional quantitative design, with intra-subject controls.

### 2.2. Participants

Participants were 98 children and adolescents (aged 7–18 years), recruited from the Outpatient Chronic Pain Clinic and the Rheumatology Clinic at Sydney Children’s Hospital between 2017 and 2019. Participants included in the study had non-neuropathic, non-cancer pain lasting longer than 3 months. Exclusion criteria included cognitive impairment, acute medical conditions, and inadequate English language. Based on the above criteria, patient eligibility was determined by the clinicians at the clinics.

### 2.3. Procedures

Ethical approval was obtained from the Sydney Children’s Hospitals Network Human Research Ethics Committee (Reference No. 13SCHN29). Written informed consent was obtained from parents and written assent obtained from participants. The SST procedure was explained and demonstrated before commencing testing. Each SST stimulus was applied in a standardized order. Stimuli were first applied at the control sites, then closely adjacent to the pain site, but avoiding the expected zone of peripheral sensitization [28]. Questionnaires were completed by the parent and participant prior to, or following, the testing.

#### 2.3.1. Questionnaires

Participants and parents were given the questionnaires summarized in Table 1.

#### 2.3.2. Somatosensory Stimulus–Response Testing

**Figure 1:**
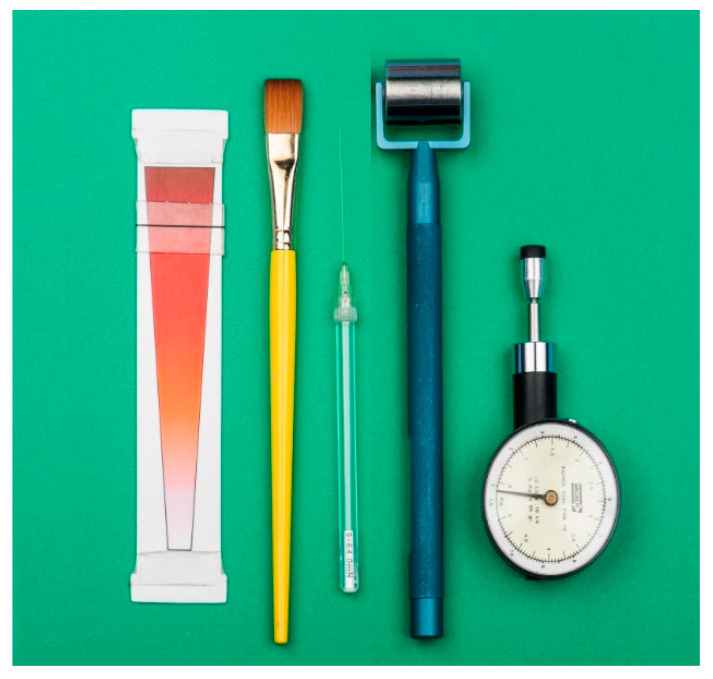
The test instruments summarised in Table 2: Coloured Analogue Scale, Camel hair brush, von Frey optical glass filament No 12, ThermoRoll (at 22 °C), Fischer Handheld Pressure Algometer. The sources are in Supplementary Table S3.

The SST protocol was derived from published QST and SST protocols and our previous pediatric studies [4,5] and is summarized in Table 2. The instruments are pictured in Figure 1 and the sources listed in Supplementary Table S3. The protocol was formulated specifically for a clinical pediatric context, taking into account portability of equipment, procedural length and acceptability for testing for children.

The procedures were performed in the order presented in Table 2, control sites first. The procedures were paused or ceased if the child exhibited any signs of distress. The intensity of responses was measured using the Coloured Analogue Scale (CAS) [33], corresponding to a 0–10 scale, with adapted anchors. The CAS is validated for use in children 5 years and older and is a reliable measure of self-reported pain [33,34]. The participant was requested to slide a marker along the scale indicating the self-assessed response within the range of no response to a very strong response for each modality (see Table 2 for specific CAS anchors used for each test).

##### Cutaneous Stimuli Responses

For all cutaneous modalities (static and dynamic light touch, single and repetitive punctate pressure, cool stimuli), control sites were the contralateral dorsum of the foot or hand, whichever was more remote from the pain site, and the contralateral cheek, determined by a literature review [35,36]. Following the applied stimuli, participants were asked to report their responses on the Coloured Analogue Scale (CAS). In a subset of 60 patients, CAS ratings were complemented by responses to a question as to whether there was pain (hurt) or not, to assess for tactile allodynia. This assessment for cutaneous allodynia was not included in the test protocol for the first 38 subjects.

##### Deep Pressure Pain Threshold via Handheld Pressure Algometry

Deep pressure response was assessed by applying the handheld Fischer Pressure Algometer (FDK 10; PDT Inc., NY, USA) with gradually increasing pressure. Participants were asked to say “Stop” when the sensation just began to hurt, at which point the pressure was released and the deep pressure pain threshold (assessed in kilograms) recorded. If the child did not say “stop” once the maximal specified pressure of 7.5 kg/cm^2^ was reached, then this maximal pressure was recorded as the deep pressure pain threshold.

Deep pressure pain threshold was assessed at control sites determined on the basis of common use in the published pressure pain literature [37,38] (opposite pain site, ipsilateral and contralateral thenar eminence or ball of big toe and dorsal forearm or tibialis anterior). This was followed by assessment of the deep pressure pain threshold at a site adjacent to, but not directly over, the main pain site.

##### Temporal Summation of Pain

For the three repetitive stimulus tests (dynamic light touch, punctate pressure and deep pressure via handheld algometry), 10 repetitions were applied at a rate of approximately one per second. If 10 repetitions were not tolerated due to the pain intensity, the number of repetitions completed was recorded. Repetitive deep pressure at the predetermined deep pressure pain threshold relevant to the site was applied at the pain site and one control site (contralateral proximal tibialis anterior). Temporal summation of pain from repetitive deep pressure was determined by subtracting a CAS score of 1 (reflecting the response at pressure pain threshold) from the CAS response after 10 repetitions.

##### Determination of Hyperesthetic and Hypoesthetic Responses

For all cutaneous responses, an abnormality score was calculated by subtracting the average CAS score at the two control sites from the CAS score at the pain site. This score was used as the cutaneous stimuli response in the analyses. Scores less than −1 were classified as hyposensitive, scores between −1 and 1 were classified as normal and scores above 1 were classified as hypersensitive. These cut offs were selected as a 1-point CAS difference has been determined as the minimum clinically significant difference [38].

##### Determination of Implied Central Sensitization Classification

Based on SST responses, and in line with the recommendations of Arendt-Neilson et al. [2], a classification of implied central sensitization (direct determination being inaccessible) determined if participants had both low (decreased) deep pressure pain threshold (via handheld algometry) and facilitated temporal summation of pain in response to deep pressure. The cut-off for low deep pressure pain threshold was set to 2.37 kg/cm^2^ based on the upper limit of normative data in children and adolescents [35], with responses below 2.37 kg/cm^2^ classified as low deep pressure pain. Facilitated temporal summation of pain was defined as the difference between the CAS score after 10 repetitions and the CAS after one stimulus being greater than 3.31 (this being the maximal observed temporal summation at the control site). Patients who did not meet both of the above criteria were classified as not having central sensitization.

##### Deep Pressure Pain Threshold and Temporal Summation of Pain via Manually Inflated Cuff Algometry

A manually inflating 13 cm blood pressure cuff was also used to assess the deep pressure pain threshold and temporal summation of pain in a subsample (*n* = 51, recruited in 2018). A single stimulation was applied by inflating the cuff at test sites (mid-upper arm or widest part of the lower leg) to determine the deep pressure pain threshold (in mmHg). The limb nearest to the primary pain site was chosen as the pain site and two control sites were the contralateral and most remote limbs. The deep pressure pain threshold was re-tested at all sites. If the participant did not say “stop” once the maximal pressure (300 mmHg) was applied, then this maximal pressure was recorded as the deep pressure pain threshold.

Dynamic stimulation was then applied with 30 s of sustained pressure at the pre-determined threshold for each site. Participants were instructed to say “stop” if the stimulation became too uncomfortable. In such cases, the seconds tolerated was recorded. The CAS rating taken after 30 s of sustained stimulation was recorded and used in temporal summation of pain calculations, including for the other modalities previously explained.

### 2.4. Statistical Analyses

The data were analyzed using SPSS software 26. Cutaneous responses were classified as hypo-esthetic, hyperesthetic or normal based on the difference between CAS ratings for the pain site relative to the average control site rating as stated above.

All of the variables were approximately normally distributed (skewness between −1 and +1; kurtosis between −3 and +3). Associations between cutaneous and deep SST responses at the pain sites, pain outcomes and biopsychosocial questionnaire responses were calculated using Pearson’s correlation coefficients.

Using the subset for which data were available for cutaneous allodynia (*n* = 60), means were compared for those individuals with at least 2 responses indicative of cutaneous allodynia (*n* = 36) relative to those individuals who did not have at least two cutaneous responses (*n* = 24), on measures of pain intensity, functional interference, depressive symptoms, pain-related anxiety and sleep impairment.

Correlational analyses (reporting Pearson coefficients) were carried out to assess the inter-relationships between cutaneous and deep stimuli responses, pain and functional outcomes, and psychosocial measures. Based on accepted convention (Cohen [39]), the magnitude of correlations was considered small (0.1), moderate (0.3) or large (0.5 or larger), whereby only moderate or large correlations were considered to be of potential clinical significance.

Multiple regression analyses were performed to determine the extent to which two criteria commonly associated with central sensitization, namely low deep pressure pain threshold and facilitated temporal summation of pain in response to deep pressure, and a self-reported measure of depressive symptoms, predicted current pain intensity and functional impairment.

Independent sample t-tests were used to compare responses of participants in the implied Central Sensitization group with those participants in the No-Central Sensitization group on a range of biopsychosocial measures.

The relative utility of cuff algometry (*n* = 51) was assessed and compared to handheld pressure algometry. Intra-rater reliability of the deep pressure pain threshold was assessed using Intraclass Correlation Coefficient (ICC) estimates with 95% confidence intervals for test–retest responses using both algometry methods at pain and control sites. Tolerability of temporal summation of pain procedure was calculated as the percentage of participants who completed either 10 repetitions for handheld pressure algometry or 30 sustained seconds for cuff algometry. Sensitivity of both algometry methods was compared through analyzing whether differences in deep pressure pain threshold and temporal summation of pain were detected between control and pain sites using paired *t*-tests.

## 3. Results

### 3.1. Sample Descriptives

The sample included 98 patients (65 females, 33 males) with chronic pain, aged 7–18 years (Mean age = 13.10 years; SD = 2.43). The duration of pain ranged from 3 months to 15 years (Mean = 3.31 years; SD = 3.51; Median = 2.0 years). The chronic pain syndromes were classified using the International Classification of Diseases (ICD-11) chronic pain classifications [40,41]: chronic secondary musculoskeletal pain (*n* = 37), chronic secondary visceral pain (*n* = 15), chronic primary visceral pain (*n* = 14), complex regional pain syndrome (CRPS; *n* = 12), chronic primary widespread pain (*n* = 8), chronic primary headache (*n* = 5), chronic secondary widespread musculoskeletal pain (*n* = 4), chronic secondary headache (*n* = 3). Patients reported an average of 5.89 pain sites each (SD = 6.11; Median = 4.00; Range: 1–33).

### 3.2. Categorisation of Normal, Hyposensitive, Hypersensitive and Allodynia Responses to Cutaneous and Deep Stimuli

Table 3 depicts the categorization of normal, hypoesthetic, hyperesthetic and tactile allodynia responses based on CAS self-reports at pain sites relative to the average of the two control sites used for each of the cutaneous tests. Hypersensitive responses were more common on all tests than hypo-sensitive responses.

### 3.3. Associations with Cutaneous Allodynia

Using the subset for whom data were collected for cutaneous allodynia (*n* = 60), it was found that those individuals with cutaneous allodynia on at least two cutaneous tests (*n* = 36) reported significantly greater pain intensity (*p* < 0.001) and greater pain-related interference (*p* = 0.03) than individuals who did not report cutaneous allodynia on at least two sites (*n* = 24). The groups did not differ significantly for depressive symptoms (*p* = 0.54), pain-related anxiety (*p* = 0.07) or sleep impairment (*p* = 0.24).

### 3.4. Deep Pressure Pain Threshold

Deep pressure pain thresholds are reported in Table 4, with an indication of the frequency and percentage of responses that were lower than the upper limit of pediatric normative data (2.37 kg/cm^2^) [35]. Of the control sites, low deep pain threshold was identified most commonly at the site opposite the primary pain site. Notably, in situations of midline or widespread pain, this control site was more likely to be affected by the pain condition. Some participants had multiple pain sites, in which case the self-reported worst pain site was used.

### 3.5. Inter-Relationships between Cutaneous and Deep Stimuli and Pain Outcomes

The Pearson correlations between all cutaneous ratings (variables 1–5 in Table 5), using the CAS, are reported in Table 5, which depicts mostly a moderate to high pattern of inter-correlations between the cutaneous SST responses. Deep pressure pain threshold was significantly negatively (albeit mildly) correlated with light touch, punctate pressure, current pain intensity and functional interference.

### 3.6. Correlations between Deep SST Responses, Pain Outcomes and Psychosocial Measures

As shown in Table 6, the self-reported psychological measures (variables 5 and 6 in Table 6) had moderate to strong correlations (based on the reporting conventions of Cohen [39]) with the pain outcome measures (variables 3, 4, 7), but with negligible or small (non-significant) correlations with deep pressure responses. The deep pressure measures (variables 1 and 2) had either no significant correlations or small correlations which were of questionable clinical significance. Functional interference correlated moderately with pain intensity and moderately or strongly with the psychological measures. Sleep impairment had a small but significant correlation with pain intensity and moderate or large correlations with the psychological measures.

### 3.7. Role of Deep SST Responses as Statistical Predictors of Pain, Functional Disability and Impaired Sleep

As shown in the Supplementary Table S4, multiple regression analyses predicting pain and impaired functioning (N = 89) revealed that self-reported depressive symptoms were a significant predictor of both dependent variables (*p* < 0.01). Deep pressure pain threshold was also a significant predictor of functional interference (*p* < 0.05), and approached significance as a predictor of pain intensity (*p* = 0.053). Temporal summation of deep pressure pain was not a significant predictor of any of the dependent variables.

### 3.8. Implied Central Sensitization Using SST and Comparison between Those with Chronic Pain Who Did and Did Not Meet the Criteria

Based on the implied central sensitization criteria, namely low deep pressure pain threshold in response to the handheld algometer and facilitated temporal summation of pain in response to repeated deep pressure, *n* = 58 (59.2%) met criteria for the implied central sensitization classification. Individuals who met the implied central sensitization criteria had on average significantly greater general anxiety, pain-specific anxiety, depressive symptoms and sleep impairment (Table 7).

### 3.9. Utility of Manually Inflated Cuff Algometry

The utility of cuff algometry (*n* = 51) for assessment of deep pressure responses was evaluated with reliability, tolerability and sensitivity analyses, and compared to handheld pressure algometry.

Strong intra-rater reliability was found between test–retest pain threshold measurements for cuff algometry (ICC: pain site = 0.831; controls = 0.856–0.918) and handheld pressure algometry (ICC: pain site = 0.927; control site = 0.780), with moderate–excellent confidence intervals at the pain site [42].

The tolerability rate of temporal summation of pain with cuff algometry was 76% at the pain site and averaged 82.3% at controls, and, with handheld pressure algometry was 69.6% at the pain site and 91.1% at control sites.

Whilst handheld pressure algometry demonstrated a significant difference between control and pain sites for pain threshold and temporal summation of pain (*p* ≤ 0.001), cuff algometry demonstrated a similarly significant difference for pain threshold (*p* ≤ 0.001), but not temporal summation of pain (*p* > 0.05). Under-powered analyses for participants with primary limb pain (*n* = 26) revealed significant differences between sites for pain threshold and temporal summation of pain (*p* < 0.05), yet no differences for participants with primary midline pain, suggesting confounding influence of primary pain site.

## 4. Discussion

In this study, we have refined the methods and extended the scope of our previous publication [4] on the utility of clinical SST with intra-subject controls in diverse chronic pediatric pain disorders. The extensions involve tactile allodynia and a composite measure of responses to deep pressure pain threshold and temporal summation in response to repetitive deep pressure at the pain threshold as indicators of mechanical hyperalgesia implying central sensitization.

The first primary aim required the initial determination of the frequency of hypoesthetic, hyperesthetic (including tactile allodynia) and normal responses to cutaneous SST stimuli at the pain site compared to control sites. The full set of von Frey microfilaments was not applied for sensory testing in this protocol, having been previously evaluated [5] and found exacting for tester and pediatric patient, especially involving several test sites and control sites. Hyperesthetic responses elicited from the pain region, and often beyond, were more frequent than hypoesthetic responses. Tactile allodynia was elicited by at least one stimulus in 81% of patients tested, most frequently by punctate pressure. In reviewing the data for deep pressure pain from multiple control sites, results were noted to be impressively consistent across the control sites, except, as anticipated, contralateral to the primary pain site where the thresholds were relatively low, but even this was not as low as at the pain site.

In line with the first primary aim, the current study found evidence of complex inter-correlations between cutaneous SST responses, deep pressure responses and biopsychosocial measures. Light touch and punctate pressure were moderately highly correlated, with slightly lower correlations with cool stimuli. The fact that correlations between deep pressure pain responses and the cutaneous test responses ranged from modest to negligible highlights that the deep pressure threshold is likely to be tapping into a different construct. There is literature to suggest that responses to deep pressure stimuli are more likely to be impacted by central sensitization [2]. It was therefore surprising that neither deep pressure pain threshold nor temporal summation of deep pressure as single measures (in some contrast with the composite measure, in next paragraph) correlated significantly with functional interference, depression, anxiety, sleep impairment, these individual measures all being commonly associated with central sensitization. Further research is needed to elucidate why these measures, all presumed to be tapping into central sensitization, were not significantly correlated.

The second primary aim was to establish the relationship between a composite measure of implied central sensitization (determined through SST), with common clinical features of central sensitization (e.g., psychological functioning, functional interference, sleep impairment). This was of importance given that of the neurobiological mechanisms involved in pain chronicity and central hyperexcitability [6,7,8,9,10,11], central sensitization is the one for which a clinician has most capacity to gain some insight and is probably most relevant. Consistent with our hypothesis, implied central sensitization was detected using the SSTs in the majority (59.2%) of chronic pain patients tested in the current study. Those assessed as having implied central sensitization had more frequent sleep impairment, generalized and pain-specific anxiety, and depression, but they did not have more severe pain intensity or functional interference. A larger study is needed to further explore the value of such a composite measure of implied central sensitization. Moreover, consideration should be given to whether cutaneous allodynia would also be a useful criterion in this composite measure.

Although the composite measure of implied central sensitization was determined through measures associated with deep pressure (namely deep pressure pain threshold and temporal summation), cutaneous allodynia may be regarded as a pain hypersensitivity phenomenon and characteristic expression of central sensitization [2] and is also a feature warranting consideration. Cutaneous (tactile) allodynia is an important symptom in chronic pain disorders, frequently distressing, and often overlooked by medical practitioners. In the current study cutaneous allodynia was most sensitively elicited by punctate pressure. Although analyses were exploratory and powered only to detect large effect sizes, individuals with cutaneous allodynia on at least two cutaneous tests reported significantly greater pain intensity and greater functional interference than individuals who did not report cutaneous allodynia on at least two sites. The groups with, versus without, cutaneous allodynia did not differ significantly for psychological symptoms or sleep impairment. Further research exploring the occurrence and relevance of cutaneous allodynia in the pediatric pain context is warranted. To date, it has been assessed in few pediatric pain studies, for example complex regional pain syndrome [43], post-surgical pain [44] and migraine [45], but not to our knowledge in miscellaneous chronic pain.

The third primary aim was to consider predictors of key pain outcome measures (functional interference and pain intensity). When considering the influence of deep pressure pain threshold, temporal summation and depressive symptoms, it was found that self-reported depressive symptoms and deep pressure pain threshold were statistically significant predictors of functional interference. However, depressive symptoms were the only significant predictor of pain intensity. These results highlight the significant role of psychological factors in the chronic pain experience. Nevertheless, deep pressure pain threshold was also found to account for a significant amount of unique variance of functional interference, suggesting that SST, particularly the measure of deep pressure threshold, may offer unique and valuable information in the clinical context to supplement other biomedical and psychological assessments.

In striving to achieve a set of somatosensory tests that are both informative and acceptable to children, a secondary aim of the current study was to determine whether a manually inflated blood pressure cuff may be used as an alternative to the handheld pressure algometer. Both methods were similarly reliable and acceptable to pediatric pain patients. Tolerability of cuff algometry was marginally higher at pain sites; however, this may have been related to the fact that the cuff was applied further from the primary pain site for children with midline pain. Cuff algometry discriminated between pain and control sites for deep pressure pain threshold but not for temporal summation of pain.

Data from the current study suggest that a manually inflated blood pressure cuff shows promise as a useful (and more readily available) alternative to the handheld pressure algometer as a method for determining pain hypersensitivity (low deep pressure pain threshold). The lack of sensitivity to detect differences between pain and control sites for temporal summation of pain likely reflected the difficulty in utilizing the blood pressure cuff at a true “pain site” for individuals with primary midline pain and insufficient statistical power. The handheld algometer may be more useable when assessing children with midline pain conditions.

Selected technical and interpretive comments might assist clinicians exploring SST. Static and dynamic light touch stimuli with an artist’s brush are standard procedures in pain orientated physical examination, not only for neuropathic but also for somatic chronic pain. Both cutaneous hypoesthesia and hyperesthesia are common in chronic somatic pain disorders [46,47], including in children [4]. Each may be present in the one individual in different distribution or at different times, and, is commonly associated with impaired tactile acuity [48]. Whilst hyperesthesia is a component of pain hypersensitivity, hypoesthesia is understood to be a plasticity phenomenon with reduced cerebral somatosensory activity, notably in the cerebral cortex, in the persistent pain context [49,50,51]. Hypoesthesia and hyperesthesia do not convey important clinical meaning to the clinician and may be considered epiphenomena, although these responses should be understood and might prove to be useful in pain phenotyping. In this study, cool stimuli by metal at 22 °C (ThermoRollm Freeport, NY USA), although frequently eliciting hyperesthesia, evoked cutaneous allodynia less frequently than mechanical stimuli.

The chosen punctate pressure instrument was a von Frey filament, OptiHair number 12, rather than pinprick for children and adolescents, and this has the advantage of stimulating mainly A*β* fibers (as opposed to pinprick) and thus potentially eliciting tactile allodynia. Overall, punctate pressure, widely accepted in QST and SST protocols, was the most sensitive static and repetitive cutaneous stimulus to elicit hyperesthetic and allodynia responses. A benign pin prick alternative would be the commercially widely available Neuropen with Neurotips (replaceable pins which, because of the spring mechanism of the Neuropen, delivers a consistent stimulus which is not usually concerning for children). The range of PinPrick stimulators (MRC QST products) is another consideration for punctate pressure. While cool stimuli were not generally informative (nor were warm stimuli by Thermoroll in a previous study [5]), occasionally cool stimuli evoke clinically relevant allodynia. Metal at controlled room temperature (22 °C) or colder can be substituted. A clinician in practice can use digital (usually thumb) pressure in place of a handheld pressure algometer. Clinicians routinely apply uniform pressure at pain and remote sites to elicit “tenderness”. Change of this technique to manual deep pressure pain threshold and facilitated temporal summation at the pain threshold relative to remote control sites would be more informative.

The application of SST methods show promise as a useful supplement to the biopsychosocial model of assessment widely used in the pediatric chronic pain context. Not only can the SST methods provide unique, clinically meaningful information, but a discussion of SST methods and results provides a pathway to engage in an informed discussion with the child and parent about the neurobiological component of the chronic pain experience. Nevertheless, there were a number of limitations in the current study. The study had a relatively small sample size, particularly with regard to the subset analyses relating to the cuff algometry and measures of allodynia and in regard to evaluating responses comparing the different pain categories. The heterogeneity of the pain disorders, particularly including midline pain disorders, reduced the sensitivity and specificity of the results, but we sought to enhance the generalizability of the SST methods by their inclusion. Another limitation was the difficulty in selecting optimal control sites for midline pain conditions such as headache and abdominal pain. Moreover, testing at clearly defined pain sites and control sites using cuff algometry was not always possible for patients with midline pain.

Although it might be suggested that the lack of external controls was a limitation, our objective was to develop and test methods applicable to research with intra-subject controls and for the methods to be applicable in clinical practice. It might also be considered that our subjects’ clinical office SST testing be compared with results from laboratory QST, but that would have been arduous for the pediatric patients and no such laboratory was regionally available. Published normative data for temporal summation of pain in healthy pediatric samples would be useful, enabling a more informed cut-off for normal and abnormal responses.

## 5. Conclusions

The SST procedures and results of this exploratory study suggests future potential applications for research and clinical practice. For research, further investigation of temporal summation to deep pressure stimuli is required with a larger sample size. A larger sample size with more restricted diagnostic categories would enable chronic pain phenotyping. A refined testing protocol may be useful to test the prediction of post-surgical and other outcomes. For the clinician, cutaneous testing with mechanical stimuli (static and dynamic/repetitive touch and punctate pressure) for allodynia is highly important and is potentially predictive of pain intensity and functional interference. Repetitive deep pressure for facilitated temporal summation of pain intensity did not add important information in this study. Tactile allodynia together with the low deep pressure pain threshold, especially when extended and accompanied by after-sensations [2], provides insight into likely central sensitization, which can alert to risk of adverse outcomes from procedural interventions, and facilitates discussion about the neurobiological dysfunction underlying chronic pain. A manually inflated blood pressure cuff may offer a useful, more readily available, tool for the assessment of deep pressure pain threshold than the handheld pressure algometer. However, the handheld algometer is likely to have greater utility for the assessment of midline pain conditions. Of the assessments relevant to the prediction of pain-related outcomes, psychosocial evaluation remains dominant.

## Figures and Tables

**Table 1:** Child and Parent Questionnaires.

Self-Reported Measure	Questionnaire	Description and Reference
Number and location of pain sites	Body map [29]	The distribution and number of pain sites in the last two weeks was recorded on a body map with a front and back view of the body demarcated into 21 regions.
Pain intensity	Faces Pain Scale—Revised (FPS-R) [30]	The FPS-R consists of 6 faces expressing increasing degrees of pain intensity corresponding to scores of 0 to 10. Validated in children above 5 years of age.
Depression, General anxiety, Pain specific anxiety	The Bath Adolescent Pain Questionnaire (BAPQ) [31]	Each subscale contains 6 or 7 questions scored on a 5-point frequency scale ranging from 0 = never to 4 = always. A composite emotional functioning measurement was also calculated by adding scores for all three subscales.
Pain related functioning	Patient Reported Outcome Measurement Informative Systems Paediatric Pain Interference Scale—Short Form (PROMIS) [32]	The PROMIS—Short Form Interference Scale consists of 8 questions assessing the effect of pain on physical, social and emotional function, scored on a 5-point frequency scale ranging from 1 = never to 5 = almost always. The PROMIS has been found to have sound psychometric properties.
Sleep Impairment	Composite measure	The composite sleep impairment score was determined as the sum of the responses to four sleep related questions from the other questionnaires.

**Table 2:** Summary of somatosensory testing methods.

Stimulus	Instrument	Method (Control and Pain Sites)	CAS * Anchors	Indicators Relevant to Pain Hypersensitivity
Static light touch	Soft artist’s brush	Brush lightly pressed onto site at 45°	“No touch” to “very strong touch” Pain yes/no	Abnormally high “touch” response Pain, highly unpleasant sensations
Dynamic light touch	Soft artist’s brush	Brush stroked in single direction ×10	“No touch” to “very strong touch” Pain yes/no	Abnormally high “touch” response Pain, unpleasant sensations, after-sensations
Punctate pressure	von Frey filament 12 **	Pressed at site until it starts to bend	“No touch” to “very strong touch” Pain yes/no	Abnormally high “touch” response Pain, unpleasant sensations
Repetitive Punctate pressure	von Frey filament 12	Pressed at site until it bends ×10	“No touch” to “very strong touch/pain” Pain yes/no	Pain, temporal summation of sensory intensity to pain
Cool Stimuli	Thermo-roller 22 °C	Rolled along skin in single direction for 3 s	“Not cold at all” to “Freezing cold” Pain yes/no	Intensely cold response Pain, highly unpleasant sensations
Deep pressure	Fischer pressure algometer	Pressure applied perpendicular to site, until begins to hurt	“No push/ pain” to “very strong push to just pain”	Low deep pressure pain threshold
	Manually inflating 13-cm blood-pressure cuff	Pressure by inflating cuff by ~10 mmHg/s, until begins to hurt	“No push pain” to “very strong push to just pain”	Low deep pressure pain threshold
Repetitive deep pressure	Fischer pressure algometer	Pressure ×10 applied at predetermined pain threshold.	CAS after 10 repetitions minus CAS at threshold (designated 1)	Temporal summation of pressure pain intensity
	Manually inflating 13-cm blood-pressure cuff	Inflating cuff 10 mmHg/s to pain threshold, maintaining for 30 s.	CAS after 30 s minus CAS at threshold (designated 1)	Temporal summation of pressure pain intensity

* Coloured Analogue Scale [33] with modified anchors. ** The von Frey optic glass filament (No 12) was chosen for punctate pressure rather than pinprick on request of Research Ethics, due to its being less concerning for children, and it also has the advantage of stimulating mainly A*β* fibers and thus is a further test for allodynia.

**Table 3:** Categorization of normal, hypoesthetic, hyperesthetic and allodynia responses to cutaneous stimuli (*n* = 98). Allodynia subset (*n* = 60).

	Normal *n* (%)	Hypoesthetic *n* (%)	Hyperesthetic *n* (%)	Allodynia *n* (%)
Static Light Touch	38 (38.8%)	24 (24.5%)	36 (36.7%)	13 (22.4%)
Dynamic Light Touch	56 (57.1%)	18 (8.2%)	24 (24.5%)	21 (36.2%)
Punctate Pressure (single)	49 (50%)	10 (10.2%)	39 (39.8%)	36 (62.1%)
Punctate Pressure (repeated)	38 (38.8%)	15 (15.3%)	45 (46.0%)	44 (75.9%)
Cool	32 (32.7%)	13 (13.3%)	53 (54.1%)	10 (17.2%)
Allodynia to at least one modality				47 (81.0%)

**Table 4:** Deep pressure pain threshold at the pain site and control sites (N = 98) and the frequency of low deep pressure pain threshold as compared with the upper limit of normative data (2.37 kg/cm^2^) [35].

Site of Stimulus Application	Pressure Pain Threshold (kg) Mean (SD)	Low Pressure Pain Threshold *n* (%)
Pain site (adjacent)	1.43 (1.40)	83 (84.7%)
Control site 1—opposite pain site	2.14 (1.46)	75 (76.5%)
Control site 2—Contralateral distal limb (thenar eminence or ball of big toe)	3.14 (1.96)	43 (43.9%)
Control site 3—ipsilateral distal limb (thenar eminence or ball of big toe distal limb)	3.25 (2.12)	41 (41.8%)
Control site 4—opposite proximal limb (dorsal forearm or tibialis anterior)	3.25 (2.14)	45 (45.9%)
Control site 5—ipsilateral proximal limb (dorsal forearm or tibialis anterior)	3.15 (2.25)	46 (46.9%)

**Table 5:** Pearson correlations between cutaneous Coloured Analogue Scale responses and deep pressure pain threshold at the pain site, as well as pain outcomes variables (pain intensity and functional interference).

	1	2	3	4	5	6	7	8
1. Static Light Touch	1	0.728 **	0.666 **	0.549 **	0.262 **	−0.211 *	−0.021	−0.059
2. Dynamic Light Touch		1	0.741 **	0.577 **	0.254 *	−0.152	0.011	−0.017
3. Punctate Pressure—single			1	0.740 **	0.400 **	−0.286 **	0.021	−0.001
4. Punctate Pressure (×10)				1	0.377 **	−0.133 *	0.011	−0.015
5. Cool					1	−0.065	−0.121	0.080
6. Deep Pressure—single (kg)						1	−0.206 *	−0.248 *
7. Current Pain intensity							1	0.321 **
8. Functional interference								1

* Sig *p* < 0.05; ** Sig *p* < 0.01.

**Table 6:** Pearson correlations between key deep somatosensory test responses, pain outcomes and psychosocial measures.

	1	2	3	4	5	6	7
1. Deep pressure pain threshold	1	−0.217 *	−0.206 *	0.020	−0.068	−0.138	0.178
2. Temporal summation of pain		1	0.027	0.150	0.202	0.236 *	0.153
3. Pain intensity (Current)			1	0.321 **	0.308 **	0.388 **	0.255 **
4. Functional interference				1	0.483 **	0.574 **	
5. Depression symptoms					1	0.697 **	0.582 **
6. Pain-specific anxiety						1	0.452 **
7. Sleep impairment							1

* Sig *p* < 0.05; ** Sig *p* < 0.01. Note that the composite measure of sleep impairment was not tested for correlation with functional interference (PROMIS) because it included sleep measures from those questionnaires.

**Table 7:** Comparison of individuals who met criteria for implied central sensitization (ICS) and those who did not.

	ICS (*n* = 58) * Mean (SD)	No ICS (*n* = 40) * Mean (SD)	*p*
General anxiety	13.58 (5.35)	10.24 (4.85)	0.003
Pain-specific anxiety	13.65 (5.43)	11.38 (5.04)	0.048
Depression	11.55 (5.19)	9.39 (4.87)	0.050
Sleep impairment	10.27 (3.53)	8.51 (2.87)	0.014
Pain intensity	3.59 (2.73)	3.03 (2.33)	0.297
Functional interference	27.69 (7.67)	25.58 (7.35)	0.201

* Numbers varied slightly for specific comparisons due to some missing data.

## Supplementary Materials

The following are available online at https://www.mdpi.com/2227-9067/7/12/275/s1, Table S1: Pressure pain threshold (PPT) in clinical samples, children and adolescents; Table S2a: QST in healthy children and adolescents; Table S2b: QST (with at least 2 modalities) in clinical samples of children and adolescents; Table S3: SST instrument sources; Table S4: Multiple regression analyses predicting pain and impaired functioning (*n* = 89 *).
