# Supplementary material for: Somatosensory Testing in Pediatric Patients with Chronic Pain: An Exploration of Clinical Utility
Source: Children (Basel). 2020 Dec 5;7(12):275. doi: 10.3390/children7120275 (PMC7762158; doi:10.3390/children7120275)
Supplement: Supplementary file 1 [file children-07-00275-s001.pdf]

Supplementary Table S1: Pressure pain threshold (PPT) in clinical samples, children and adolescents.

First author	Reference	Age (yrs)	Disorder	Results
Alfvén G	Acta Paediatr 1993; 82:481-3	11	Recurrent abdominal pain	PPT significantly lower in abdominal wall near umbilices in those with abdominal pain than controls. PPT also lower in 5 other muscle sites.
Hogeweg JA	Pain 1995; 62: 11-7	6-17	Juvenile chronic arthritis	Lower PPT at inflamed joint sites and at remote (paraspinal) sites.
Hogeweg JA	Br J Rheumatol 1995; 34: 61-7	11.5 ±3.1	Juvenile chronic arthritis	Low PPT interpreted as evidence for central hyperexcitability.
Reid GJ	Arthritis Rheum 1997; 40:752-60	14.6 ±1.89	Primary juvenile fibromyalgia	Low PPT at tender points compared to control points, low mean PPT at control points
Duarte MA	J Pediatr Gastroenterol Nutr 2000; 31: 280-5	5-15.8	Recurrent abdominal pain	PPT lower in abdominal wall and at 4 other sites than in controls
Buskila D	Arch Pediatr Adolesc Med 2003; 157:1079-82	12-18	Prematurely born infants	PPT lower in premature born children than controls. Most did not report pain.
Hashkes PJ	J Rheumatol 2004; 31:610-3	4-12	Growing pains	PPT lower at multiple lower limb and other sites.
Metsahonkala L	Eur J Pain 2006; 10: 581-5	13	Migraine and tension headaches	No significant differences in PPT at extracephalic muscular sites for migraine and headaches compared with controls.
Chaves TC	J Orofac Pain 2007; 21:133-42	7-12	Temporomandibular pain	Reliability study of algometer. Satisfactory results.
Jedel E	Eur J Pain 2007; 11:557-63	12.1±1.7	Temporomandibular pain	No significant contrasts with controls
Hermann C	Pain 2008;135:397-406	12.0±1.5	Recurrent abdominal pain	Low PPT interpreted as evidence for central hyperexcitability.
Zohsel K	Eur J Pain 2008; 12:1090-101		Migraine	Results interpreted as evidence of central hyperexcitability.
Fernandez-de-las-Peñas C	Cephalalgia 2010; 30: 1049-55	8.9-1.8	Headache (tension type)	Bilateral widespread pressure pain hypersensitivity compared to controls
Nikolajsen L	J Child Orthop 2011; 5: 173-8	4-12	Orthopaedic disorders	PPT methods using algometry. Good reliability and well tolerated.
Soee AB	Cephalagia 2013; 33:454-62	7-17	Headache (tension-type)	Pressure pain sensitivity by method of levels (5 increasing intensities) showed increased pain sensitivity compared with controls (VAS) at trapezius and temporalis muscle sites.

Leegard A	J Rheumatol 2013; 40: 1212-7	13-18	Juvenile idiopathic arthritis	PPT lower at multiple sites (joint and bone) compared with controls. Girls had lower PPT than boys.
Ferracini GN	Pain Med 2014; 15: 702-9	6-12	Migraine	Pericranial and cervical sites had lower PPT than extra-cephalic sites and lower PPT than controls.
Winger A	BMJ Open 2014;4: e005920	12-18	Chronic fatigue syndrome	One-hundred and twenty adolescents with CFS and 39 healthy controls. Patients with CFS had significantly lower PPTs compared with controls.
Rathleff MS	Clin J Pain 2016; 32: 428-34	15-19	Patellofemoral pain	PPT used in controlled trial.
King CD	Clin J Pain 2017; 33:620-626	15.4±1.4	Juvenile Fibromyalgia	Thirty-four females with JFM and 31 controls. Adolescents with JFM had greater sensitivity to pressure pain compared to controls.
Sa S	Musculoskelet Sci and Pract 2017; 30:18-24	16-18	Chronic idiopathic neck pain	Adolescents with neck pain showed higher levels of catastrophizing and anxiety, lower pressure pain thresholds and higher joint repositioning error than asymptomatic controls. Pain intensity, frequency and duration were moderately correlated with anxiety, and disability was moderately correlated with anxiety and catastrophizing.
Scheper MC	Arthritis Care Res 2017; 69(3):421-429	<18	Ehlers-Danlos Syndrome Hypermobility type (EDS-HT)	Significantly lower pressure-pain thresholds were found in children with hypermobility syndrome (HMS) and Ehlers-Danlos syndrome hypermobility type compared to normative values. The presence of generalized hyperalgesia discriminated between individuals with HMS/EDS-HT, hypermobility, and healthy controls (odds ratio 6.0).
Nahman-Averbuch H	Pain 2019; 160(5): 1019-1028	12-17	Migraine	Pain inhibition was assessed by conditioned pain modulation (CPM), which used both suprathreshold heat pain (heat CPM) and pressure pain thresholds (pressure CPM) as the test stimuli before and during cold-water immersion. Adolescents with migraine and healthy adolescents have similar inhibitory pain modulation capability, despite having marked differences in pain sensitivity.
Pas R	Pain 2019; 160: 1883-90	6-12	Functional abdominal pain disorders (FAPD)	Compared with pain-free controls, young children with FAPD showed lower pressure pain thresholds (PPT) at all test sites, a lower conditioned pain response to cold pressor task, more functional disability, and pain-related fear.
Valentino R	J Oral Rehabil 2020; 47 (8): 944-950	M 12 SD 3	Juvenile Idiopathic Arthritis (JIA)	Pressure pain thresholds were significantly lower among JIA patients compared with controls for all analysed sites. The presence of TMJ pain at palpation was significantly associated with a lower pressure pain threshold at TMJ.

Campi LB	J Oral Facial Pain Headache 2020; 34(1):83-91	12-14	Painful temporomandibular disorders (TMD)	Significant associations between signs of painful TMD and the number of tender points, as well as between tender points and the pressure pain threshold values for local, regional, and widespread pain, were found. No association between signs of painful TMD and fibromyalgia was found.

Supplementary Table S2a: QST in healthy children and adolescents.

First author	Reference	Age (yrs)	Results
Hilz MJ	J Clinical Neurophysiol 1998; 15: 529-34	7-17.9	Normative data for warm and cold perception thresholds using the method of limits at the volar distal forearm, thenar eminence, lower medial calf, lateral dorsal foot, and cheek. There was good reproducibility of thresholds. Preference for large probe where it adjusts planely.
Meier PM	Muscle Nerve 2001; 24: 1339-45	6-17	Cold, warm, heat vibration detection thresholds determined by methods of limits and levels. Both methods well accepted and reproducible.
Lu Q	J Pain 2007	8-18	Testing included coping predictors of laboratory-induced pain tolerance, intensity and unpleasantness. Also assessed pressure, heat and cold stimuli. Catastrophizing predicted higher pain intensity and higher cold pain unpleasantness, Seeking emotional support predicted lower pain tolerance. Methods of levels applied.
Blankenburg M*	Pain 2010; 149: 76-88	6-12	Younger children less sensitive to thermal and mechanical detection stimuli but more sensitive to pain stimuli. Girls more sensitive to thermal detection and pain stimuli, but not to mechanical detection and pain stimuli. Face more sensitive than hand, foot. QST feasible and valid for children over 5 years with their own reference values.
Blankenburg M*	Pain 2011; 152: 2625-31	7 and 14	7-year-olds more sensitive than 14-year-olds to most pain stimuli except cold pain, but same as 14 year olds in mechanical and thermal detection thresholds except cold. Girls more sensitive only to warm detection.
Hirschfeld G*	Neuropediatrics 2012; 43: 10-16	6-16	QST parameters showed good short-term reliability. Over 1 year children became less sensitive to pain stimuli, but no difference over 1 year in non-nociceptive somatosensory processing.
Boerner KE	Pain 2014; 155: 983-93		Review of publications on sex differences in experimental pain (cold-pressor, heat pain, pressure pain). Meta-analysis showed girls reported higher for cold-pressor pain when older than 12 years, and heat pain tolerance.
van den Bosch GE	BMC Pediatrics 2017;17:77:1-10	8.2-17.9	Seventy-one Dutch children, thermal QST only. The protocol is feasible. Females more sensitive for both cold and heat, the youngest children (8-9 years) were less sensitive to warm stimulus.
Dua K	J Pediatric Orthopaedics 2019; 39(2):98-103	2-17	Monofilament and static/moving 2-point discrimination tests were performed bilaterally assessing the median, ulnar, and radial nerves to determine reliability and normative values. For the monofilament test, 27% of 3-year-olds, 83% of 4-year-olds, and all participants 5 years of age and older were capable of performing the monofilament test. For 2-point discrimination tests, 33% of 4-year-olds, 61% of 5-year-olds, 88% of 6-year-olds, 95% of 7- and 8-year-olds, and all participants 9 years and older were capable of performing the static/moving 2-point discrimination tests.

* German Research Network on Neuropathic Pain.

Supplementary Table S2b: QST (with at least 2 modalities) in clinical samples of children and adolescents.

First author	Reference	Age (yrs)	Disorder	Results
Abad F	Diabetes Med 2002; 19: 827-31	8-16	Diabetes I neurologically asymptomatic of diabetes	Increased thresholds for warmth in hand, cold and warmth in foot. At least one abnormality in 43%. Correlation study for heat pain threshold and duration.
Zohsel K	Pain 2006; 123: 10-18	9-15	Migraine	Enhanced sensitivity to painful stimuli at trigeminal site (mechanical not heat) in those with migraine of average duration 4.4 years, especially girls. Heat and mechanical pain thresholds higher when mother present.
Sethna NF	Pain 2007; 131: 153-61	13.2± 2.6	Complex Regional Pain Syndrome	Cold, heat and mechanical (static and dynamic) allodynia were tested. Thermal and mechanical sensory abnormalities appeared in different combinations in different patients despite similar clinical presentations. Results consistent with pain of central origin.
Schmelzle-Lubiecki BM	Eur J Pain 2007; 11:799-809	9-12	Early infant injury (cardiac surgery)	Reduced sensitivity to thermal and mechanical stimuli including non-injured (thenar) area.
Zohsel K	Am J Gastroenterol 2008; 103: 1517-23	10.7± 1.7	Recurrent abdominal pain	QST results interpreted as indicating central hyperexcitability.
Walker SM	Pain 2009; 141: 79-87	11.2±0.04	Children born extremely pre-term (intensive care and surgery)	Generalized decreases in thermal sensitivity but not in mechanical sensitivity interpreted as suggesting centrally mediated alterations in the modulation of C-fibre nociceptor pathways.
Wollgarten-Hadamek I	Pain 2009; 141: 165-72	9-16	Burn injuries in infancy	Higher mechanical detection thresholds (thenar) and lower mechanical pain thresholds, greater perceptual sensitization to repetitive mechanical stimuli. Increased thermal pain threshold only after severe burns.
Kristensen AD	Br. J. Anaesth 2012; 109:603-8	7.8 ± 2.6	Post inguinal hernia repair	Testing 3.2 ± 1.3 years after surgery. Pinprick hyperalgesia and decreased pressure pain thresholds on operated side in all 3 children tested (participants selected due to pain in inguinal region).
Blankenburg M	Diabet Med 2012; 29: 1425-32	13.2 ± 2.5	Diabetic neuropathy	Almost half of the 45 children had QST evidence of (subclinical) large- and small- neuropathies. Tactile hypoesthesia had the highest discriminatory values.
Cornelissen L	Pediatr Rheumatol Online J 2014; 12: 39.	7-17	Juvenile idiopathic arthritis	Generalised multimodal (pressure, touch, cold, heat pain) hypersensitivity at the thenar eminence, even in the absence of pain reports or markers of disease activity.

Stabell N	J Pain 2014; 15: 898-9-6	Mean=16	Irritable bowel syndrome	Multiple side lower heat and pressure pain threshold in IBS compared with controls after adjustment for psychological distress, independent of sex and comorbid pain.
Jacob E	J Pediatr Haematol Oncol 2015; 37: 185-9	10-17	Sickle cell disease	Thirteen of 48 participants showed evidence of disordered somatosensory processing: decreased sensitivity to heat or cold. Some had hypersensitive responses, even allodynia.
Tham SW	Pain 2016; 157: 2807-2815	15-19	Chronic pain regions	Chronic regional pain in 197 compared with controls 744. Responses to cutaneous thermal stimuli and to deep pressure stimuli (pain threshold and tolerance) at trapezius and finger. Lower pressure pain threshold and tolerance in chronic pain cases at trapezius.
Barney CC	Am J Intellect Dev Disabil 2017; 122(5):409-421	4.9 $\pm$ 1.13	Global developmental delay (GDD)	Case control to modified QST, reactivity determined by behavioural coding across vocal, facial and gross motor responses. On average the children with GDD were significantly more reactive than controls to most tactile sensory modalities including light touch, pin prick, cool, pressure, and repeated von Frey.
Moshourab R	Sci rep 2017; 7(1):4251	14-20	Congenital deafness	Subjects with congenital deafness were characterized by significantly higher vibration detection thresholds at 10 Hz (2-fold increase) and 125 Hz compared to controls. These sensory changes were not accompanied by any major change in measures of pain perception.
Bakshi N	J Pain Res 2018; 11: 435-43	8-21	Sickle Cell Disease (SCD)	Anxiety, depressive symptoms, catastrophizing, and somatization were found to be associated with increased sensitivity to experimental pain stimuli. Increased frequency of painful episodes in SCD was associated with decreased sensitivity to heat pain and decreased mechanical temporal summation.
Blankenburg M	Eur J Paediatri Neurol 2018; 22: 470-481	6-20	Cerebral Palsy (CP)	Thirty CP patients compared with age/sex matched controls. CP patients less sensitive to all mechanical and thermal stimuli. Fifty percent of CP patients had mechanical hypoesthesia, thermal hypoesthesia and mechanical hyperalgesia; 67% of CP patients had PVL, which was correlated with mechanic and thermal hypoesthesia
Riquelme I	J Abnorm Child Psychol 2018; 46(8):1731-1740	4-15	Autism spectrum disorder (ASD)	Children with high-functioning ASD were randomly assigned to either the intervention or control group. The intervention group received somatosensory therapy. Children in the intervention group showed a significant reduction of pain sensitivity and increase of tactile sensitivity after treatment, whereas children in the control group displayed increased pain

				sensitivity in the absence of changes of tactile sensitivity. The repetitive somatosensory stimulation therapy led to a decrease of pain sensitivity and an increase of tactile sensitivity.
Schoth DE	BMJ Open 2019; 9: e031861	6-24	Paediatric Chronic Pain	Proposed protocol for a systematic review and meta-analyses of association between QST and pain or disability.
Gulewitsch MD	Pain Med 2019; 20(8): 1472-1478	8.5-16	Functional abdominal pain	Children/adolescents with abdominal pain (AP) showed a divergent reaction regarding their sensory threshold after social exclusion. The increase of both thresholds ("numbing") after Cyberball (which affects an experience of social exclusion) was positively correlated with symptoms of mental health issues.
Miller R	British Journal of Haematology 2019; 185(5): 925-934	8-20	Children with sickle cell disease	The study provides preliminary data to support the future examination of QST as a potentially valuable outcome measure for use in early phase clinical trials to indicate response to a preventative pain therapy.
Lieber S	Eur J Pain 2018; 22 (3): 457-469	6-18	Survivors of pediatric Acute lymphoblastic leukemia	Cross-sectional, bicentric study of 46 participants using bedside Pediatric-modified Total Neuropathy Score, QST, and Nerve conduction studies. In QST, the survivor group showed significant ($p < 0.001$) inferior large-fiber function and pain sensitization when compared to healthy matched peers.
Teles AR	The Spine J 2019; 19(4):677-686	10-21	Adolescent idiopathic scoliosis and chronic back pain	Efficient pain inhibitory response was observed in 51.1% of patients, while 21.3% and 27.7% had sub-optimal and inefficient conditioned pain modulation (CPM), respectively. Temporal summation of pain was observed in 11.7% of patients. Significant correlations were observed between deformity severity and pain pressure thresholds and CPM, neuropathic pain scores and pain pressure thresholds and temporal summation of pain, and heat temperature threshold and pain intensity.
Payne LA	Pain 2019; 160(6): 1421-1430	16-24	Primary dysmenorrhea	QST results indicated enhanced pain sensitivity in young women with primary dysmenorrhea as measured by heat pain tolerance and Average Pain50, compared with healthy controls. These group differences were evident at all phases of the menstrual cycle. No group differences in cold pain tolerance, Temporal summation, or conditioned pain modulation were evident at any phase of the menstrual cycle.
Silva KE	Ann Clin Transl Neurol 2019; 6(2): 344-354	13.8±5.6	Children with posterior cerebellar tumour resection – low grade gliomas	Twelve pediatric patients and twelve matched controls evaluated with QST and fMRI were evaluated for pain processing. Minor abnormalities only.

Brandow AM	Pain 2019; 160(2):407-416	7-19	Sickle cell disease (SCD)	Patients underwent quantitative sensory testing during baseline health and within 48 hours of hospitalization for acute pain. Patients with SCD had increased cold pain sensitivity in the hand during hospitalization compared with baseline and increased mechanical pain sensitivity in the foot during hospitalization. There were no differences in heat pain sensitivity.
Hainsworth KR	Children 2020; 7: 55	Adolescents	Chronic pain and obesity	Adolescents with chronic pain and obesity had higher thermal and mechanical pain thresholds than controls with pain only or obesity only.

Supplementary Table S3: SST instrument sources.

Instrument	Note / Brand or Make
Camel hair brush	Alternative: or other soft artist`s brush
Calibrated von Frey monofilament No 12	VFI 2: set of 12 optical glass filaments. Formerly Marstock, now MRC, Germany
Metal ThermoRoll	Formerly Marstock, now Rolltemp II, Somedic SenseLab, Sweden
Fischer pressure algometer	FDK 10; PDT Inc, NY, USA

Supplementary Table S4: Multiple regression analyses predicting pain and impaired functioning (n = 89 *).

Dependent variable: Current Pain Intensity	Standardized B	t	p
Deep Pressure Pain Threshold	-.200	-1.96	.053
Temporal Summation of Pain (for deep pressure)	-.045	-.434	.666
Depressive symptoms	.303	2.940	.004

Dependent variable: Functional Interference	Standardized B	t	p
Deep Pressure Pain Threshold	-.219	-2.349	.021
Temporal Summation of Pain (for deep pressure)	.037	.389	.698
Depressive symptoms	.463	4.886	.000

* Regression analyses are reported for n = 89 because self-reported depression scores were missing for n = 9.
